# Overestimating the Self, Outranking the Group: An Experimental Study of Overconfidence Biases in Young Decision-Makers

**DOI:** 10.3390/bs15121671

**Published:** 2025-12-03

**Authors:** Duygu Güner Gültekin, Funda Nur Akıncı

**Affiliations:** 1 School of Business and Management Sciences, İstanbul Medipol University, Istanbul 34815, Türkiye; 2 Faculty of Education, Yıldız Technical University, Istanbul 34165, Türkiye; fnakinci@yildiz.edu.tr

**Keywords:** overconfidence bias, overplacement, overestimation, cognitive biases

## Abstract

Cognitive biases have been proved to have a systematic influence on decision-making at both individual and social levels. This study investigated two forms of overconfidence—specifically, overestimation and overplacement—among young individuals participating in a social prediction task using an experimental design. The experimental study was conducted with 414 undergraduate students during an in-class written exam. Participants were drawn from different courses taught by the same instructor and varied in terms of their level of prior interaction with the instructor (having attended one, two, or three semesters). Participants predicted the instructor’s favourite songs and subsequently evaluated the accuracy of their own predictions and those of their classmates. This experimental design allowed the researchers to look at both how accurately participants judged themselves and how they compared themselves to their peers. The analysis primarily focused on discrepancies between self-evaluated and actual performance. Results revealed a consistent pattern of overestimation and overplacement. Participants rated themselves as more successful than they actually were and positioned themselves above the average of their peer group. Moreover, the lack of direct feedback and limited contextual cues in the guessing task created a psychological ambiguity, which may have contributed to an unjustified sense of certainty among the participants. These findings offer empirical insight into the functioning of cognitive biases and contribute to a nuanced understanding of overconfidence in young decision-makers.

## 1. Introduction

During decision-making processes, individual cognitive biases frequently influence people’s strategic and daily decisions. When making decisions under uncertainty, people often resort to intuitive rules; although these rules are quick and economical, they can lead to systematic and predictable errors (Tversky & Kahneman, 1974). In this context, the way individuals assess their own abilities and knowledge levels also stands out as an important area of bias. One of the most common and influential cognitive biases in this area is how individuals evaluate not only their own abilities and confidence but also those of others, which may lead to systematic misjudgements in self-other comparisons. This is a process central to the study of confidence judgments and self-assessment. The modern conceptualization of the realism of trust emerged in the scientific literature through research conducted in the mid-20th century. Specifically, studies on the methodology of assessing confidence judgments (e.g., Adams & Adams, 1961), which formed the normative framework for modern calibration research, were quickly followed by important empirical studies demonstrating the decoupling of confidence from objective performance in professional mental health decisions (e.g., Oskamp, 1965). Subsequently, this empirical foundation was expanded by probabilistic assessment studies (e.g., Alpert & Raiffa, 1982) that demonstrated underestimation of uncertain quantities. As a result, this line of research, focusing on the lack of realism in self-assessment, laid the empirical foundation for future studies of the phenomenon identified as overconfidence bias. According to the literature, overconfidence (OC) is a fundamental bias, defined as the perception that one’s knowledge or abilities exceed objective performance (Moore & Healy, 2008). This bias appears in three distinct forms: overestimation, overplacement, and overprecision (Moore & Healy, 2008). Overestimation and overplacement are closely related because both result from subjective perceptions of competence (Larrick et al., 2007). However, despite their theoretical differences, applied research and indirect measures often treat overconfidence as a single construct due to the difficulty in reliably distinguishing these dimensions (Kommalapati, 2023). Moore and Healy (2008) classify calibration bias as an aspect of overconfidence and define it as the systematic mismatch between how individuals assess their own knowledge or skill levels and their actual performance; it encompasses the tendency to be overconfident in difficult tasks and underconfident in easy tasks. It is important for measuring the extent to which subjective probability estimates in decision-making correspond to objective accuracy (Suantak et al., 1996).

Beyond its conceptual and methodological definitions, overconfidence is recognized as a powerful behavioural force with significant practical and evolutionary consequences. Overconfidence is defined as individuals perceiving their own abilities higher than they actually are and emerges as an evolutionarily advantageous strategy in situations of uncertainty and high stakes (Johnson & Fowler, 2011); this may explain the behaviour of humans and other organisms to demand resources in competitive environments and not to retreat from conflicts. The importance of this investigation is underscored by cross-country evidence demonstrating that men’s relatively higher overconfidence is the largest behavioural factor explaining gender gaps in preferences for economic outcomes, such as redistribution choices (Buser et al., 2020). Furthermore, the literature demonstrates the influence of cognitive biases in professional decisions. Specifically, these findings suggest that managers should recognize the limitations of their own knowledge and judgment and that OC can influence decision-making (Russo & Schoemaker, 1992). Building on this, Berthet (2022) emphasizes that OC is the most common bias affecting decisions in fields such as management, finance, medicine, and law. Similarly, a comprehensive review by Rau and Bromiley (2025) indicates that overconfidence can influence decision-making in professional settings, sometimes providing motivation or strategic advantages. Additionally, empirical studies also indicate that overconfidence can lead to systematic misjudgements that influence outcomes (Ben-David et al., 2007; Zhou et al., 2025). Taken together, this highlights the broader relevance of overconfidence not only in professional settings but also in everyday social decision-making contexts.

Understanding these biases matters for both theory and practice in education, management, and daily life. Self-assessment mechanisms, though not the main focus, can offer a clear lens for observing overconfidence in real-world tasks. Building on this, a gap remains: most studies examine overconfidence and its subtypes separately, and few addresses these biases in social contexts. Many studies confirm these biases across mechanisms and variables, but few examine them empirically in social settings. Research links overconfidence to factors like gender and social comparison (Buser et al., 2020; Dahlbom et al., 2011; Croson & Gneezy, 2009) but rarely analyses both overestimation and overplacement together in low-information social inference tasks. Many studies on the three forms of self-confidence biases either examine these phenomena in isolation or analyse participants’ individual assessments of their performance through general competence tests. However, in decision-making processes occurring in social contexts, particularly in prediction and estimation tasks with limited information and no immediate outcome feedback, may reveal systematic biases. In such cases, individuals’ perceptions of their own and the peers’ success may be affected. The literature offers limited empirical findings on how these tasks intersect with individual self-evaluation and social comparison tendencies. This study aims to observe overestimation, overplacement, and overprecision biases within the same experimental design by analysing how individuals evaluate their own and others’ success in social prediction tasks. The focus is on young decision makers, specifically Generation Z students enrolled in higher education. Instead of comparing ages, we explore how confidence biases appear in this distinct, influential cohort. This is important because confidence biases can vary by age. For example, Dahlbom et al. (2011) found that boys displayed overconfidence about their math grades, while girls were underconfident. This challenges the generalized findings from adult populations. Focusing on this group is further justified by evidence that overestimation biases are highly prevalent among undergraduates, especially those in social science and business (Mahmood, 2016). By examining this cohort, the study provides a developmental perspective on these cognitive biases before individuals enter high-stakes professional settings.

In this context, the study aims to contribute to the literature by bringing a holistic approach to the triple overconfidence framework and examining the relevance of these cognitive biases within educational practices. In this way, the study seeks to address both the emergence of cognitive biases in a social context and their manifestations in young adults.

In this context, the study seeks to answer the following research questions:

1. Does the difference between participants’ estimated individual success and their actual success indicate a tendency toward overestimation?
2. Do participants systematically overplace themselves when evaluating their position within a social group?
3. Do these biases vary by the gender of the participant?
4. Do these biases vary by the familiarity with the instructor?

### 1.1. Literature Review

#### 1.1.1. Overestimation, Overplacement, and Overprecision

According to Moore and Healy (2008), there are three forms of overconfidence: overestimation, overplacement, and overprecision. Overestimation occurs when people over value their own ability, performance, or chances of success; where overplacement (also called better-than-average effect) occurs when individuals perceive themselves as superior to others in terms of their abilities or performance. Overprecision refers to the tendency to show excessive certainty in personal judgments and decisions. These concepts can be illustrated as follows: if individual X believes s/he answered 6 questions correctly on a 10-question test, but actually answered only four, this is overestimation. If s/he thinks her/his score is higher than most of the class where actually only half scored better, this is overplacement. If s/he is 90% confident in her/his answers, but the correct answers rarely fall within this estimated range, this is overprecision. Such overconfidence appears in classic studies in which people provide judgmental fractals for uncertain quantities. For example, Alpert and Raiffa (1982) found that, for a standard interquartile range (25th to 75th percentile), the true value fell outside this range much more often than the expected 50%, providing early and strong evidence for systematic overprecision. Building on this, Soll and Klayman (2004) argue that this miscalibration is not mainly due to statistical noise or variability but instead results from basic cognitive biases, such as confirmation bias and limited sampling of evidence, which lead to unjustified certainty.

This example illustrates that people possess limited insight into their own performance and even less into others’, which generates varying patterns of overestimation and overplacement based on task difficulty. These findings are initially supported by Oskamp’s (1965) analysis of psychologists’ diagnostic overconfidence and Svenson’s (1981) assessment of students’ driving skills. In Oskamp’s study, participants completed increasingly complex case studies; although their confidence grew as they received more information, their diagnostic accuracy barely improved. In short, gaining information did not guarantee more accurate judgments. Similarly, in Svenson found that, most American and Swedish students rated their driving skills as above average, considering themselves as safer and more competent than the typical driver, despite these beliefs not mirroring classroom performance. These examples reveal that individuals recurrently display overconfidence and rate their abilities above their actual performance, even when given additional information. Overprecision can also affect how individuals are perceived in group contexts, as overconfident individuals may appear more competent than they actually are, thus influencing group judgments (Moore et al., 2015).

#### 1.1.2. Overplacement and Calibration Bias

While opinions and abilities may seem distinct, they shape our behaviour together. In particular, how a person views their situation (their beliefs and opinions) along with their assessment of their own capabilities, jointly determine behaviour (Festinger, 1954). This relationship is illustrated by a well-known example: Svenson’s (1981) study examining the confidence levels of American and Swedish students regarding their driving skills. In this study, 88% of Americans and 77% of Swedes believed they were safer than the median driver. Additionally, 93% of Americans and 69% of Swedes regarded themselves as more skilled than the median driver.

These findings suggest that people not only think they are better than they really are, but also see themselves as better than others, a phenomenon known as overplacement. This tendency can be directly linked to the example of Individual X mentioned above, who misjudged both his own performance (overestimation) and his relative position in the group (overplacement). Here, social comparison becomes a critical mechanism that reinforces overconfidence, as individuals construct their self-perceptions by comparing their abilities to those of others rather than relying on objective performance. Overplacement has been found to significantly differentiate career success. Buser et al. (2021) and Niederle and Vesterlund (2007) show that a preference for competition—a mechanism linked to overplacement—independently predicts educational and labour-market outcomes, including income and field of study. Fallucchi et al. (2020) find that this preference can be measured with a single experimentally validated, non-incentivized survey item and that deep competitive preferences transfer across contexts. They stress that competition is a relevant psychological trait and a reliable predictive force.

Moreover, this phenomenon is closely related to calibration bias. This bias makes individuals overly confident (overestimating their chances of success) in difficult tasks and underconfident (underestimating their competence) in easy ones. Suantak et al. (1996) showed that systematic calibration error, not random errors, drives this bias. People often misjudge how their confidence aligns with actual performance. They may ignore contradictory evidence and focus on supporting reasons, leading to overconfidence even when memory is accurate (Koriat et al., 1980). For example, students in educational settings may feel unjustified confidence in hard exam questions and act too cautiously in simple ones, distorting their judgments of knowledge and abilities. Lundeberg et al. (1994) found that men tend to be overconfident in incorrect answers, while women often underestimate their own abilities even when they perform well. This means that self-confidence judgments can be influenced by gender, task difficulty, performance, and vary across individuals. Recent research further confirms systematic differences across socio-demographic factors, showing that, men exhibit higher levels of overconfidence than women, particularly in competitive environments (Buser et al., 2020; Dahlbom et al., 2011; Croson & Gneezy, 2009). This gender difference in subjective perceptions, where men are consistently more overconfident than women, is a critical mechanism; Buser et al. (2020) find that this higher male confidence strongly mediates the gender gap, leading men to favour significantly less redistribution when income is earned through performance.

This study examines Gen Z undergraduate students and their performance in a social guessing task, providing empirical evidence on the manifestation of overconfidence, overplacement and calibration biases in a social-skill context, and depicts the relationship between self-perception and actual performance.

#### 1.1.3. Cognitive and Social Mechanisms of Overconfidence

Cognitive mechanisms, such as the availability heuristic and confirmation bias, contribute to individuals overestimating their abilities. One of the most important factors in poor calibration is the tendency to draw overly consistent evidence from memory, a form of confirmation bias. This leads decision-makers, such as assessors or forecasters, to construct confidence intervals significantly narrower than those justified (Soll & Klayman, 2004). This narrowing effect leads them to fail to fully account for the uncertainty and variability inherent in their own judgments, thereby weakening self-assessment. Social mechanisms, such as peer comparison and status considerations, further strengthen the overplacement tendencies. Understanding these mechanisms helps contextualize patterns of overconfidence observed in educational and professional settings (Moore & Cain, 2007). These mechanisms are also related to the Dunning-Kruger effect (Kruger & Dunning, 1999): low-competence individuals tend to misjudge both their own and others’ performance. This effect offers an additional perspective in explaining the social and cognitive mechanisms of overconfidence and self-evaluation.

Overconfidence, overplacement, and calibration bias reveal the role of self-assessment mechanisms in shaping judgments and actions. Individuals make decisions based on their own assessments in daily life. Therefore, self-assessment guides our understanding of the congruence between individuals’ self-perceptions of performance and objective performance. Zell and Krizan (2014) also stated that factors affecting the accuracy of self-assessment include, the specificity and timing of questions, familiarity, complexity and objectivity of performance tasks, and the extent to which performance tasks are self-directed. Meta-analytic evidence suggests that self-assessments are only moderately accurate. However, accuracy increases when assessments are domain-specific, familiar, low-complexity, or based on objective tasks (Zell & Krizan, 2014). This inaccuracy is widely documented in educational settings, where studies frequently report overconfidence among freshman students and a negligible correlation between self-reported confidence and actual test scores (Gustavson & Nall, 2011). These findings are consistent with the Dunning–Kruger effect, which suggests that individuals with low competence tend to inaccurately assess both their own and others’ performance (Kruger & Dunning, 1999). This widespread lack of calibration is also a problem in academic contexts; studies with undergraduate students consistently demonstrate that self-confidence does not equate to research competence. In fact, high self-confidence is often unrelated to actual accuracy (Molteni & Chan, 2015). Amirkhanyan et al. (2021) further indicate that social context significantly moderates the extent of overconfidence, reinforcing the need to examine these biases in group settings where peer comparison is implicitly or explicitly available. Specifically, regarding social consequences, recent work that differentiated communication channels found that verbally expressed overconfidence in managerial contexts leads to lower brand trust and product evaluation, indirectly supporting the status-diminishing effect observed by Tenney et al. (2019) rather than status enhancement (Hilgert, 2020). Understanding these mechanisms helps explain why self-perceptions often differ from objective performance and how cognitive and social factors jointly influence overconfidence.

#### 1.1.4. Generation (Gen) Z and Overconfidence

Generation Z includes people born from 1995 to 2012 (Jayatissa, 2023; McKinsey & Company, 2024; Wibowo et al., 2023). They are the second-youngest generation, between millennials and Generation Alpha. The digital age, climate anxiety, shifting finances, and COVID-19 have shaped Gen Z’s identity.

Gen Z is an especially relevant group for examining overconfidence. They grew up in a digital world of constant social comparison and visible performance. This can widen the gap between how good they think they are and their actual ability. Their formative years also saw more uncertainty—economic turbulence, the pandemic, and global crises. Such conditions foster a need for control and a tendency toward exaggerated certainty. As a digitally fluent group, Gen Z often sees quick access to information as expertise. These may make them prone to specific types of overconfidence (McKinsey & Company, 2024). A study in Vietnam shows that overconfidence directly affects Gen Z stock investment decisions. Gen Z investors consistently overestimate their knowledge, underestimate risks, and trade more frequently and aggressively (Bich et al., 2023). Participants claimed they understood market trends, could time trades, and assess risks well, but this confidence did not match their actual knowledge. The study shows that overconfidence is a key behavioural bias shaping Gen Z financial choice. Syukur et al. (2025) also note that younger generations are more prone to cognitive biases—specifically, overconfidence, availability heuristics, and the Dunning-Kruger effect. Because they are highly exposed to digital tools and social media, these individuals tend to access information quickly, which in turn creates a perception of “I already know.” This dynamic may pave the way for overconfidence. Wibowo et al. (2023) found that overconfidence among Gen Z in Malang City weakened the quality of investment decisions and lowered risk perception. Similarly, Fahlin and Gustafsson (2024) observed that Gen Z showed stronger overconfidence in investment behaviour than Generation X. Generation Z has been shaped by widespread digital technologies and ongoing exposure to social media platforms (Twenge, 2017). Frequent social comparisons and self-presentation pressures in curated online environments may influence their self-perception and judgment (Taylor & Armes, 2024). Socio-technical factors such as algorithm-driven feedback and constant peer visibility are thought to reinforce self-enhancement motives (Chou & Edge, 2012), thereby increasing overplacement or other forms of overconfidence. Accordingly, the present study uses this cohort not merely as a demographic descriptor but as a theoretically relevant population for exploring how context-specific cognitive biases manifest in younger adults.

Building on this, while existing studies have focused on investment behaviour, research examining Gen Z’s confidence in their real-world social abilities remains limited. In our case, students often implied that they were highly skilled at detecting and interpreting subtle social cues from someone they had met with at least once a week for three months. This assumption motivated the present research design. Consistent with patterns reported in investment-related contexts, the results revealed that they were more confident in their abilities than warranted.

## 2. Materials and Methods

### 2.1. Participants

Participants were 414 undergraduate students enrolled in the Faculty of Business and Management Sciences at İstanbul Medipol University. They were recruited using a purposive convenience sampling method, as only students enrolled in the courses taught by the author (as the instructor) were invited to participate. Participation was voluntary and took place during the final exams of the spring term (2024–2025 academic year). However, the instructor was not present during the written exams (several proctors supervised that process) when the data were collected. Of the 591 students present for the exams, 414 voluntarily responded to the bonus question, which did not affect their course grades. Participation was voluntary, and the instructor’s absence helped minimize social desirability effects and implicit pressure. These steps are designed to support participants’ independence in their responses. Students were offered extra credits for their final exam scores as an incentive. Participation and completing the experimental questions fully earned 5 bonus points, with an additional 5 points granted if their guesses were correct. This gradual incentive structure was designed to increase the need for accuracy on the decision makers’ side.

During data cleaning, responses that were missing or lacked complete answers to the bonus question were excluded. The final analytical sample included 414 valid cases, each with a fully completed task. The participants in the study were primarily freshmen in their second term, accounting for 44% of the total. Among the participants, 55.8% were men and 44.2% were women. More than 72% of the participants originated from Türkiye, while the remaining participants came from various countries, including Turkmenistan, Tunisia, Syria, Pakistan, Iran, Palestine, Russia, Algeria, and Somalia. All participants were members of Gen Z, and the primary focus was on examining self-evaluative biases within this cohort.

### 2.2. Procedure

The experimental task was inspired by in-class discussions and student debates. These students reported high confidence in their ability to interpret instructors’ preferences and subtle social cues. To minimize memory-based priming and prevent direct associations with prior classroom discussions, data collection was conducted during the final examination period. This occurred several weeks after the relevant interactions. The task was framed as a non-academic guessing activity—specifically, predicting the instructor’s favourite song. This helped to establish psychological distance from the academic evaluation context. The methodology was intended to reduce demand characteristics and encourage participants to rely on spontaneous interpersonal judgment.

The entire experiment was completed within 3 days, with all participants present in the designated experimental (exam) halls. Participation was voluntary, and students could choose to opt in after receiving an explanation of the study’s purpose and procedures. Each student took a 3-item social guessing quiz. The main task of the participants was to guess and name a song they believed the lecturer would like. Then to guess their probability to hit the correct answer (for overestimation), the percentage of others who guessed right (overplacement). The participants’ guesses were evaluated and coded by the lecturer according to their accuracy (100 if the written song was actually one of the lecturer’s liked songs; 0 if not).

The task was designed to elicit participants’ intuitive judgments under uncertainty in a socially contextualized setting. Tversky and Kahneman (1974) contended that significant decisions frequently rely on beliefs regarding the likelihood of uncertain events, such as election outcomes, a defendant’s guilt, or future currency values. Their framework for decision-making under uncertainty emphasizes incomplete knowledge, unpredictable outcomes, and reliance on heuristics. Lipshitz and Strauss (1997) categorized types of uncertainty according to issues, referring to what the decision maker is uncertain about, and sources, indicating the origins of this uncertainty. The three primary issues are outcomes, situation, and alternatives, while the three main sources are incomplete information, inadequate understanding, and undifferentiated alternatives. In alignment with Tversky and Kahneman (1974) and Lipshitz and Strauss (1997), the present study defines ‘uncertainty’ as a decision-making context characterized by incomplete information, unpredictable outcomes, or the inability to assign precise probabilities. In our study, participants tried to guess the instructor’s musical preferences without any clear hints or previous examples. Because they did not get immediate feedback, their success depended on their own judgment. Although the task was challenging, it effectively captures epistemic uncertainty, making it well-suited for studying judgment biases such as overconfidence. Inspired by prior studies that incorporate personal experience into confidence tasks (e.g., Soll & Klayman, 2004), our design differs in that it draws on students’ relational familiarity with the instructor rather than factual or spatial knowledge. This structure allowed us to observe overconfidence biases in a naturalistic, low-stakes environment while preserving key cognitive dimensions of self-assessment.

In the studies concerning overplacement participants often asked to rate themselves compared to an unknown, imaginary average person (Burson et al., 2006; Ehrlinger et al., 2016; Hofer et al., 2022). However, such comparisons can be dangerous as they may reinforce group stereotypes, like “California drivers are better drivers.” To mitigate this threat Svenson’s (1981) experiment required subjects to compare themselves to a specific group of drivers with identifiable characteristics. In line with the rationale from Svenson’s (1981) study, we asked students to assess their confidence levels by comparing themselves to a clearly defined peer group. The peer groups were defined as the classmates enrolled in the same course with the participant.

### 2.3. Ethics Statement

Ethical approval was obtained from the Ethics Committee of İstanbul Medipol University (Approval No: 279, Date: 20 May 2025). All participants were informed about the voluntary nature of the study and assured of the confidentiality and anonymity of their responses.

### 2.4. Statistical Considerations

Although an a priori power analysis was not conducted, a post hoc sensitivity analysis with G*Power (version 3.1.9.7) showed that a sample of 352 would suffice to detect a small-to-medium effect size (d = 0.30) with 80% power (α = 0.05). The final sample of 414 exceeds this, indicating adequate power, even for non-parametric tests.

The collected data were planned to be analysed using descriptive statistics and comparative tests such as t-tests and correlation analysis to assess overconfidence levels. The overestimation score, calibration error, and overplacement gap were calculated using the formulas detailed in Section 2.5. Further statistical analysis details will be provided in the Section 3.

### 2.5. Variables and Operationalization

The measures utilised in the study are presented in Table 1.

### 2.6. Derived Variables

When a student rates their probability of success high but fails to correctly guess the song, this indicates overestimation, which reflects a positive discrepancy between perceived and actual performance. In contrast, calibration bias refers to the absolute difference between the self-assessed probability and actual accuracy—regardless of whether the estimate was too high or too low. This calculation approach is consistent with previous research on overconfidence, which has used raw difference scores to estimate bias (e.g., Binnendyk & Pennycook, 2024; Moore & Healy, 2008).

Overestimation Score = b − a > 0 (underestimation if b − a < 0)

Calibration Bias = |b − a|

Overplacement gap = b − c

Overplacement = (b − c) − (a − c′)

### 2.7. Scope and Generalizability

This study does not claim universal results. The findings are specific to one educational setting and are influenced by the group’s cognitive and motivational characteristics. The interpretations are limited by cultural, institutional, and demographic factors, along with methodological issues like non-random sampling and using just one behavioural task. These are common challenges in behavioural research. We see these limitations not as flaws, but as factors that set the boundaries for how our results can be understood.

## 3. Results

Initial analyses revealed non-normal distribution for key variables such as accuracy rates, overconfidence, and peer expectations. Therefore, non-parametric statistical methods were employed for hypothesis testing, including Kruskal–Wallis tests for group comparisons, Mann–Whitney U tests for pairwise comparisons, Wilcoxon Signed-Rank Test to see individual differences and Spearman’s rank-order correlations.

### 3.1. Descriptive Statistics

A total of 414 valid participation forms were obtained and analysed. The sample included 183 females and 231 male undergraduate students at School of Business and Management Faculty.

Peer expectations are slightly higher among women (36.2) compared to men (M = 33.3), though this difference is statistically insignificant (*p* = 0.810).

Both groups tend to overestimate their own achievements relative to their actual performance: the average overestimation for men is 19.8 points, compared to 15.9 points. The difference is small and statistically insignificant (*p =* 0.687), but the trend is slightly higher for men. Calibration error, which represents the absolute discrepancy between self-assessment and actual accuracy, is somewhat greater among male participants (51.6) than female participants (47.4). However, this difference also fails to reach statistical significance (*p* = 0.317).

In terms of overplacement, male participants report a modest positive gap (+0.44), while females show a slightly negative average (−0.57). Again, this variation does not indicate a significant group-level difference (*p* = 0.857), but the direction of the values may suggest a subtle gendered pattern, with men exhibiting a marginally higher tendency to view themselves as above average. Descriptive statistics are presented in Table 2.

### 3.2. Overestimation Bias

**Figure 1:**
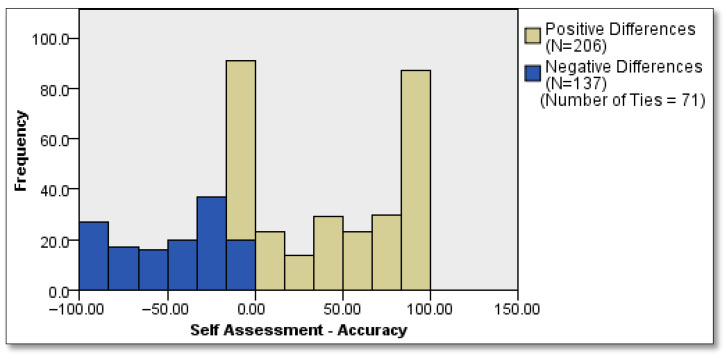
The Wilcoxon signed-rank test results.

The mean self-assessment scores between male and female participants are quite similar—approximately 63 points. There are also small differences in accuracy. However, the standard deviations indicate that the values vary considerably across individuals, highlighting inter-individual heterogeneity. The Wilcoxon signed-rank test revealed a statistically significant difference between participants’ self-assessment scores and their actual performance (accuracy), *Z* = 6.003, *p* < 0.001. This result indicates that, overall, participants significantly overestimated their own performance. Among 414 paired observations, 206 showed positive differences (i.e., self-assessment exceeded actual performance), whereas only 137 showed negative differences (underestimation). Additionally, 71 cases had no difference (ties). The distribution of differences, as visualized in the histogram, further supports the conclusion that overestimation was a prevalent pattern in the sample. The effect size was calculated as r = 0.295, indicating a moderate practical impact of the observed overestimation bias. The details are presented in Figure 1.

Participants had varying degrees of familiarity with the researcher, which is assumed to relate to the length of their interactions. About 62% of the participants had taken one course with the researcher as the instructor, consisting of 14 weeks of lectures followed by 3 weeks of exams. Furthermore, 21% of the participants had completed two courses, while 17% had taken three courses, leading to increased interaction with the instructor. However, familiarity with the instructor did not significantly affect any of the measured variables, including accuracy, suggesting that prior acquaintance with the instructor did not introduce systematic bias in participants’ self-assessments or influence performance outcomes.

### 3.3. Group-Level Comparisons for Overestimation

b − a ≤ −10 → 1: Underconfident

−10 < b − a < +10 → 2: Well-calibrated

b − a ≥ +10 → 3: Overconfident

A chi-square test revealed a statistically significant association between confidence level and prediction accuracy (χ^2^(2) = 329.69, *p* < 0.001). The underconfident participants were uniformly accurate, whereas overconfident individuals consistently failed in their predictions. Well-calibrated participants showed a mixed outcome. This strong alignment supports the notion that overconfidence is associated with poorer actual performance, whereas underconfidence may reflect cautious but accurate self-assessment. Details are presented in Table 3.

**Figure 2:**
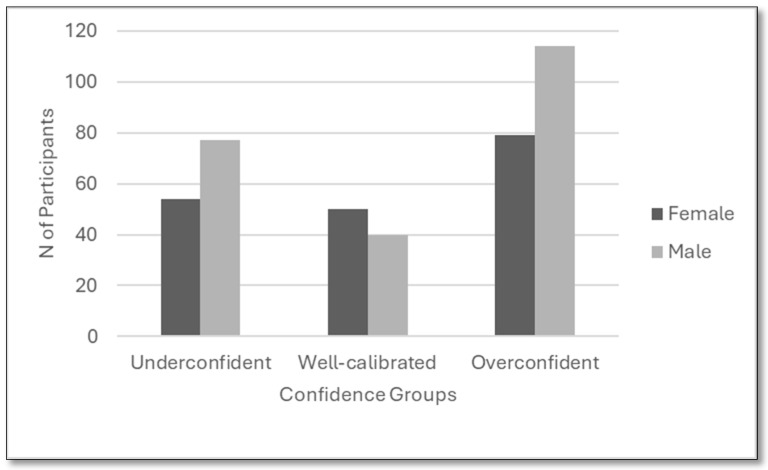
Gender distribution across confidence groups.

A chi-square test of independence revealed a statistically significant relationship between gender and confidence calibration group (χ^2^(2) = 6.01, *p* = 0.049). While men were more likely to be categorized as overconfident (59%) or underconfident (58%), women were slightly more represented in the well-calibrated group. This suggests subtle but significant gender-based differences in self-assessment accuracy. Details are presented in Figure 2.

A total of 128 participants made correct predictions and expressed more than 50% confidence in their choices. In contrast, 114 participants indicated less than 50% confidence in their freely chosen answers. Among these 114 participants, 51 provided the correct answer, demonstrating underconfidence. From a statistical decision-making perspective, self-reported probabilities of success below 50% typically suggest that the decision-maker believes their choice is more likely to be incorrect than correct. In normative decision-making models this probability structure would imply that revising the initial response could increase the likelihood of earning more credit. Yet, despite signalling underconfidence, these participants seem not to choose to change their initial answers.

### 3.4. Overplacement

The foundational study for this research is Moore and Healy (2008). Unlike their design, which asked participants to estimate the performance of a randomly selected previous peer, the present study required participants to form expectations about the performance of their current classmates. For analytical purposes, students were grouped into five distinct peer groups, each corresponding to a different class section. Participants estimated the average prediction success rate of their own peer group.

Consistent with the computation outlined by Moore and Healy (2008), a corrected overplacement score was calculated to account for both perceived and actual performance differences between individuals and their peers. The formula applied was as follows:

Overplacement = (b − c) − (a − c′)

In this formula, b denotes the participant’s belief regarding their own expected performance, c indicates their belief about the expected performance of their peer group, and a and c′ represent the participant’s actual score and the actual mean score of their peer group, respectively. Unlike the original study, which utilized a Randomly Selected Previous Peer (RSPP), the present study employed actual peer group averages as the comparison benchmark. This methodological adjustment enables isolation of the overplacement component attributable to cognitive bias rather than actual performance differences.

**Figure 3:**
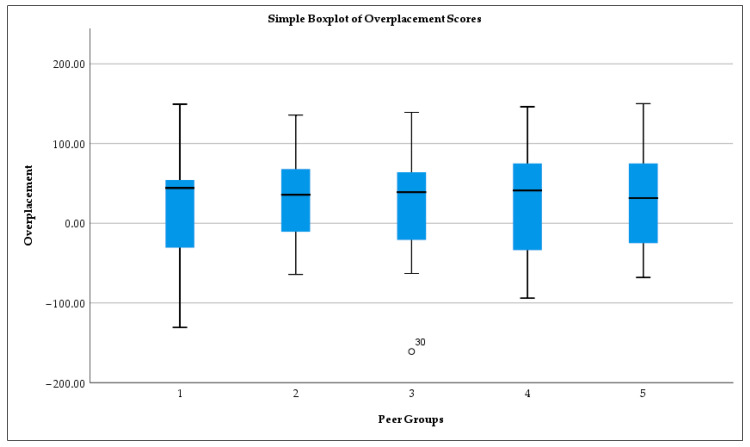
Overplacement score across peer groups.

Among these peer groups, the lowest mean self-assessment score was 61, the lowest mean peer expectation score was 32, and the lowest mean accuracy score was 35. Despite these descriptive differences, no statistically significant differences emerged among the peer groups for any of the three variables. However, the overall results demonstrate a consistent pattern: participants perceived themselves as more successful than their actual performance and as superior to their peers. The details are presented in Figure 3.

A generalized linear model with peer group, gender, and familiarity with the instructor as fixed factors did not produce statistically significant effects on overplacement scores (Omnibus Test: χ^2^(7) = 2.38, *p* = 0.936). None of the main effects were significant: peer group (*p* = 0.846), gender (*p* = 0.359), or familiarity (*p* = 0.530). These findings indicate that these variables did not account for the observed variability in overplacement.

### 3.5. Calibration Curve and Overplacement Gap

**Figure 4:**
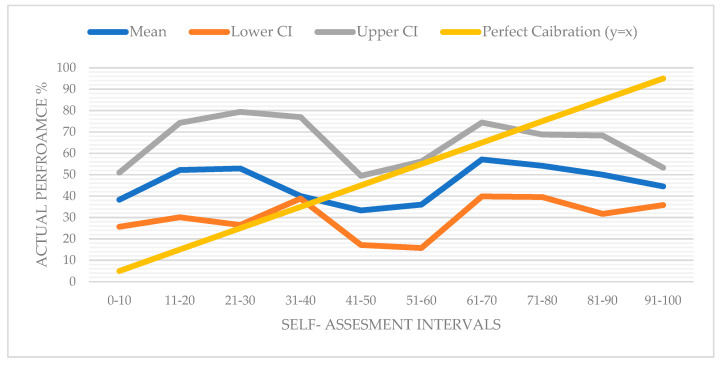
Calibration curve.

The calibration curve (displayed in Figure 4) illustrates the relationship between self-assessment intervals and corresponding average accuracy scores. While a perfectly calibrated individual would exhibit a 1:1 correspondence between perceived and actual performance, the observed pattern deviates from this ideal. While some intervals (e.g., 31–40 or 61–70) show relatively accurate self-judgments, others exhibit substantial misalignment—particularly in the mid-range (41–60). This miscalibration pattern suggests the presence of overconfidence bias, wherein perceived performance systematically exceeds actual outcomes.

The calibration curve reveals a noteworthy pattern. Individuals with lower self-assessments appear to have underestimated their performance, as their accuracy values lie above the reference line. In contrast, another group with similar performance levels overestimated their abilities, with their accuracy falling below the perfect calibration line. Since the latter group is more numerous, the overall calibration curve tends to fall below the reference line—reflecting a general trend of overconfidence across the sample. This descriptive pattern is statistically supported by a Spearman correlation analysis, which found no significant relationship between self-assessed and actual performance scores (ρ = 0.033, *p* = 0.506). This further reinforces the misalignment between participants’ confidence levels and their objective performance, indicating a general lack of calibration. A regression analysis was performed to evaluate the effect of self-assessment on accuracy. The model was not statistically significant (F(1, 412) = 0.77, *p* = 0.380), indicating that self-assessed confidence did not significantly predict actual performance. The explained variance was minimal (R^2^ = 0.002), and the coefficient for self-assessment was not significant (B = 0.062, *p* = 0.380), with a 95% confidence interval that included zero [−0.076, 0.199]. These findings indicate no systematic relationship between confidence and accuracy.

A Mann–Whitney U test was conducted to examine whether participants’ actual performance (accuracy) differed significantly based on their self-assessed probability of success, categorized as below or above 40%. The results indicated no statistically significant difference between the groups in terms of accuracy (M = −50.55, U = 63,484, *p* = 0.663). On average, participants with low self-assessments (<40%) scored 44.5% on the accuracy measure, while those with higher self-assessments scored 45.4%. However, there was a significant difference in overplacement gap (M = 18.30, U = 16,316, *p* < 0.001). The average overplacement score was −50.6 in the low self-assessment group and +18.3 in the high self-assessment group. Participants with higher self-assessments exhibited a markedly greater tendency to over-place themselves relative to their peers, despite showing comparable levels of actual performance.

A generalized linear model revealed that confidence group significantly influenced overestimation, with underconfident, well-calibrated, and overconfident participants showing notable differences (χ^2^(2) = 1379.49, *p* < 0.001). However, gender (χ^2^(1) = 0.20, *p* = 0.653), familiarity with the instructor (χ^2^(2) = 0.39, *p* = 0.824), and peer group (*p* = 0.846) showed no significant effects on either overestimation or overplacement. Furthermore, interaction effects between confidence group and either gender or familiarity, as well as the omnibus test for all factors (χ^2^(7) = 2.38, *p* = 0.936), were non-significant, indicating that these variables did not explain the observed variability in overestimation and overplacement.

We also conducted Spearman correlation analyses to examine the relationships between overestimation, overplacement, and calibration error. Results showed positive correlations among all three measures. Overplacement was significantly associated with overestimation (ρ = 0.869, *p* < 0.001) and also with calibration error (ρ = 0.309, *p* < 0.001). These findings suggest the three indicators are related, but each captures a distinct aspect of miscalibration.

Overall, the findings show consistent evidence of overconfidence in judgment. Participants overestimated the likelihood of their guess being correct and believed they outperformed their peers. These patterns support both overestimation and overplacement biases. The results affirm Research Question 1, demonstrating a clear pattern of overestimation bias: across 414 paired observations, participants were far more likely to overrate their performance (206 cases) than to underestimate it (137 cases), indicating a systematic inflation of self-perceived ability relative to actual outcomes. A significant association between confidence level and prediction accuracy observed: the underconfident were uniformly accurate, overconfident consistently failed in their predictions. For Research Question 2, the gap between self- and peer-assessment confirms the overplacement tendencies. Strikingly, participants’ actual performance (35) aligned far more closely with the level they expected from their peers (32)—a level they explicitly undervalued—than with the level they expected from themselves (61). This pattern suggests a pronounced miscalibration, in which participants not only overestimated their own abilities but also underestimated others’. Furthermore, addressing Research Question 3, the analysis revealed no significant gender differences in the degree of overplacement, indicating that both male and female participants displayed comparable levels of judgment bias. Turning to Research Question 4, the results showed that students with longer exposure to the instructor (i.e., across 2 or 3 semesters) exhibited slightly lower levels of overestimation, suggesting a limited but observable priming effect due to familiarity with the instructor’s expectations. Together, these findings contribute to a nuanced understanding of the dynamics of overconfidence in novice decision makers.

## 4. Discussion

This study aims to assess overconfidence bias by comparing individuals’ self-efficacy perceptions with their actual performance in a social guessing task. Findings indicate a significant overestimation bias across the sample. Ayton and McClelland (1997) argued that confidence levels often do not match actual accuracy. Our findings support this: participants’ self-assessed confidence and actual performance did not correlate significantly. This reinforces that overconfidence can persist without reliable metacognitive insight.

Although overestimation was stronger, a clear overplacement bias also emerged. Participants rated their expected performance (M = 61) much higher than their peers’ (M = 32), even though their actual performance (M = 35) was close to the peer estimate. This gap underscores a robust overplacement effect: participants both overvalued their abilities and undervalued others’. These results echo earlier research on comparative judgment errors (Kruger, 1999; Moore & Healy, 2008), suggesting comparative biases are driven by perceived rather than objective competence (Larrick et al., 2007). Even in ambiguous contexts, individuals maintain inflated self-assessments, as Kommalapati (2023) notes about measuring overconfidence. However, this strong pattern of overplacement misaligns with previous studies suggesting that people exhibit lower levels of overplacement when they lack domain familiarity or clear performance standards (Kruger, 1999; Burson et al., 2006).

The calibration curve (Figure 3) illustrates the discrepancy between participants’ self-assessments and their actual performance. The largest deviations occur within the middle confidence intervals, indicating that individuals are more prone to calibration errors when making intermediate predictions than when expressing high or low self-confidence. Spearman’s correlation (*p* = 0.506) indicates no significant association between self-confidence and actual success. This decoupling of confidence from actual success illustrates poor metacognitive monitoring, a key feature in decision-making under uncertainty (Schraw, 2009; Kleitman & Stankov, 2007). However, the validity of assessing such subjective biases using self-reported probabilistic estimates remains strong; experimental validation confirms that non-incentivized survey items can accurately measure core preference for competition, suggesting that these responses capture a fundamental, portable psychological trait (Fallucchi et al., 2020). The calibration curve further demonstrates systematic differences between predicted and actual success rates, particularly among overconfident participants.

Building on previous research that used personally relevant estimation domains to assess confidence calibration (Soll & Klayman, 2004; Peon et al., 2014), our study takes a novel approach. We shift the focus from analytic estimations to socially embedded inferences. In our task, students made plausible guesses based on their ongoing exposure to the instructor’s personal cues. This approach activated their intuitive reasoning and placed overconfidence in an interpersonal context. Our design offers a novel contribution to the overconfidence literature by including social cognition, relational memory, and informal decision-making. These areas are often underexplored in traditional studies of overconfidence.

These findings can be considered broadly consistent with the overconfidence literature, and they show some parallels with patterns described in the Dunning–Kruger effect. Kruger and Dunning (1999) showed that low-competence individuals misjudge their own and others’ performance. Their self-confidence may inversely relate to actual achievement. These patterns are consistent with metacognitive deficits observed in low-ability individuals, as outlined by Dunning (2011), who emphasized the dual challenge of poor performers being unaware of their deficiencies. In our study, similar patterns emerged—namely, overconfident individuals exhibited low accuracy but high self-confidence, while underconfident participants were more accurate yet less confident. Specifically, the fact that individuals in the underconfident group had low self-assessment scores despite achieving 100% accuracy in their predictions (Table 3) suggests a different pattern. This finding aligns with the literature indicating that miscalibration may transition to underconfidence at very high levels of competence (Lichtenstein & Fischhoff, 1977). This pattern illustrates a type of miscalibration in which relatively competent individuals systematically underestimate their own abilities. This bipolar manifestation of miscalibration, encompassing both the overconfidence of low performers and the lack of confidence of high performers, can be interpreted as a significant gap in metacognitive awareness even among high-performing young decision-makers. Nevertheless, we did not conduct curvilinear analyses to formally test for this misalignment. Therefore, any reference to the Dunning–Kruger effect should be interpreted as illustrative rather than as empirical confirmation.

The estimation task used in this study can be considered difficult because it involves low information and requires subjective judgments based on subtle social cues. Indeed, the participants’ actual success rate was only 35%, indicating that the task involved cognitive demand and ambiguity despite the lack of explicit uncertainty. Research in the literature indicates that the tendency to overestimate becomes stronger as task difficulty increases, and that individuals systematically overestimate their success on difficult tasks (Suantak et al., 1996; Koriat et al., 1980; Larrick et al., 2007). As emphasized in Moore and Healy’s (2008) overconfidence framework, individuals are more likely to overestimate their own performance, especially in uncertain and feedback-free situations. The findings of this study suggest that this mechanism operates similarly in a social estimation context: participants significantly overestimated their own performance in a challenging, low-information social task. In this respect, the present findings not only confirm the difficulty-overestimation relationship in cognitive estimation and probability assessment studies but also demonstrate that it holds true in the context of social decision-making. Thus, the inherent uncertainty and social agency of the task suggest that calibration errors documented across various fields of the literature are also reflected in everyday social judgments among young adults. This result offers an important contribution to the literature by demonstrating that the tendency to overestimate exhibits a systematic pattern not only in academic or analytical tasks but also in social cognitive processes. Participants’ low accuracy (35%) in predicting another person’s preferences is consistent with social comparison theory (Festinger, 1954), which posits that people are frequently mistaken when predicting the thoughts and tendencies of others, and suggests that this cognitive bias is also evident in social inference tasks among Gen Z. This study’s findings align with the emerging literature, showing that young adults’ self-confidence levels can deviate from their actual achievement. Participants reported an average confidence of 57% but only achieved a 35% success rate. This outcome supports Kleka et al. (2019), who demonstrated that metacognitive awareness is only beginning to develop in this age group. Similarly, these young decision makers tend to overestimate their own success relative to their peers—although not statistically significant—is consistent with Phi Huynh et al.’s (2025) findings highlighting the impact of social comparison on academic self-concept, suggesting that estimates made at low knowledge levels can often be biased by upward comparisons in the social context. Turning to gender differences, males reported higher self-confidence, but their performance did not surpass females. McMichael and Kwan (2025) observed that although young men expect greater success than women, this expectation often fails to translate into better performance.

The overplacement gap is significantly greater among individuals with higher self-assessment scores, although actual performance does not differ significantly, as indicated by the Mann–Whitney U test. Figure 2 presents the average accuracy levels for each self-confidence group and demonstrates a positive association between underconfidence and high achievement. This pattern indicates that high self-confidence may coexist with low competence, as noted by Dunning (2011). Specifically, the underconfident group achieved an average accuracy of 100%, while the overconfident group achieved 0%. The well-calibrated group attained approximately 65% accuracy (Table 3). Additionally, 51 participants exhibited self-confidence below 50% despite providing correct answers. These results suggest that students can make accurate decisions even when lacking confidence.

Concerning peer expectations, the Kruskal–Wallis test reveals a statistically significant difference among the confidence groups (*p* = 0.032). However, post hoc pairwise comparisons, adjusted for multiple testing, do not reveal any statistically significant differences between specific groups.

Although overall gender differences were not statistically significant, descriptive trends suggested a slight positive bias among male participants. Men showed a positive bias in overplacement scores (+8.86), while women had a slight negative bias (−0.57). This pattern may suggest that men tend to view their performance as superior to their peers, whereas women tend to have more modest self-assessments. Chi-square analyses indicated that gender differences were present across self-confidence calibration groups, with male participants tending to be overconfident. This result aligns with the literature, which shows that women generally have lower self-confidence than men, yet their performance can be higher. For example, a study conducted in the context of medical education found that women performed better on the OSCE exams, yet their self-confidence levels were significantly lower than those of men (Bodard et al., 2024). Furthermore, in STEM fields, it was observed that while female students achieved higher scores than their male counterparts, they reported lower self-efficacy perceptions (Whitcomb et al., 2020). Barber and Odean (2001) similarly found that men exhibit more overconfidence in financial decision-making. Similar gender-based discrepancies were observed for highly competitive decision-making environments (Buser et al., 2020; Dahlbom et al., 2011; Croson & Gneezy, 2009).

Together, these findings reinforce the presence of overconfidence and overplacement biases, rather than a rational alignment between subjective expectations and objective results.

Informal interviews following the experiment indicated that students employed heuristic strategies when selecting songs, such as choosing those they believed corresponded to the instructor’s age group. However, participants did not seem to utilize similar strategies when making probabilistic predictions. These findings suggest a limited ability among students to integrate statistical reasoning into intuitive decision-making processes. This may be attributed to the domain-specific nature of heuristics. As Gigerenzer (2007) posits, heuristics can serve as efficient decision tools under bounded rationality; however, their effectiveness often depends on familiarity with the context. When faced with probabilistic reasoning tasks, especially in unfamiliar or abstract domains, individuals may lack transferable strategies and default to intuition or guesswork.

It is important to note, however, that the single-trial, context-free estimation task used in this study differs methodologically from the multiple, context-focused measures common in this literature. This distinction requires careful interpretation regarding the generalizability of the findings.

## 5. Conclusions

The experimental task was designed based on informal observations and discussions with students during the academic term. Many participants reported strong confidence in their ability to intuit the preferences and dispositions of instructors and peers. This remained true even without explicit cues or prior knowledge. This pattern, especially evident among Generation Z students, indicated a form of interpersonal overconfidence. Specifically, it was the belief that one can accurately interpret others in low-feedback social situations. Rather than generalizing overconfidence across all decision-making domains, the present study targeted this specific, socially situated judgment bias in a controlled experimental context. Although the task format may appear artificial, it is meant to model a psychologically valid phenomenon from educational settings.

The findings of this study demonstrated that overconfidence can also be effectively observed in the context of social comparison. The findings indicated that participants generally exaggerated their achievements (overestimation) and tend to view themselves as more successful than their peers (overplacement). The reverse calibration pattern, in which the underconfident group makes 100% correct predictions and the overconfident group makes completely incorrect ones, is a finding rarely seen in the decision-making literature and represents a unique contribution to the context of social prediction.

Furthermore, participants consistently underestimated their peers’ performance (%32); interestingly, this underestimated expectation was actually very close to the group’s average prediction accuracy (35%). This pattern suggests that the cognitive distortion lies not in their perception of others, but in the inflated self-appraisal that places their own performance substantially above both peers and reality. This asymmetric miscalibration reinforces the overplacement bias: participants correctly gauged the group’s low performance yet unjustifiably believed themselves to be exempt from it. Participants overestimated their own achievements while largely accurately estimating their peers’ performance, suggesting that the overplacement bias observed in this study primarily resulted from self-overvaluation rather than from underestimating others. This finding partially contradicts previous research, which suggests that individuals tend to misjudge both their own and others’ performance (e.g., Kruger, 1999; Burson et al., 2006).

The calibration curve and statistical analyses revealed that participants’ self-assessments systematically mismatched their actual achievements. This mismatch was particularly pronounced at mid-range confidence intervals, suggesting low levels of metacognitive awareness. This deviation indicates that people are more likely to be influenced by cognitive biases when they feel most uncertain during social prediction tasks and also it supports the yet-to-be-developed self-awareness of young adults. These patterns align with existing research on metacognitive calibration, which suggests that individuals with low competence may lack the insight needed to accurately monitor their own performance (Schraw, 2009; Dunning, 2011) and the young adults are still in the process of developing their self-awareness to its full potential (Kleka et al., 2019). In this context, the study’s findings suggest that self-confidence can be both exaggerated at low competence and under expressed at high competence, consistent with the Dunning-Kruger effect. Additionally, the observed 1:1 deviation in the calibration curve indicates that the overprecision bias documented in previous research is also present in social forecasting contexts (Alpert & Raiffa, 1982; Soll & Klayman, 2004).

The fact that participants’ prediction accuracy and their familiarity are not correlated partially contradicts previous studies suggesting that familiarity improves calibration (Zell & Krizan, 2014). On the other hand, the relatively weak gender differences in this study may suggest that gender-based overconfidence effects, which are widely reported in the literature (Buser et al., 2020; Dahlbom et al., 2011; Croson & Gneezy, 2009), are significantly weaker in the context of social forecasting.

In conclusion, this study, though limited by sample and scope, highlights that social contextual judgments in young adults are vulnerable to cognitive biases. This suggests that overestimation and overplacement are not just analytical phenomena but also arise in low-information social inference. These insights imply that overconfidence may broadly shape social cognition in young adults, though the findings are context specific. Although overconfidence is a well-documented cognitive bias, the present study makes a novel contribution by examining it within a naturalistic, low-information task in an authentic educational context. Given the study’s single-task design and reliance on self-reported estimates, these findings should be interpreted as exploratory.

## 6. Limitations and Future Research

This study has several limitations. First, the sample originated from a single university and faculty, which limits its generalizability. The experimental task, guessing the instructor’s favourite song, also occurred in a specific context that allowed participants to develop personal strategies. Most participants were 18–22 years old. Future studies should include different age groups to test how self-confidence and calibration errors vary across the lifespan. Experience, self-awareness, and decision-making strategies developed over time may influence overconfidence. Thus, future research should include diverse demographic groups.

Students received extra points for correctly guessing the song, which may have affected motivation and performance. While the gradual bonus point system may have introduced motivational engagement, it primarily aimed to encourage thoughtful participation rather than manipulate cognitive judgment and was consistent with real-world classroom incentives. However, rewarding not only correct guesses but also accurate probabilistic predictions could encourage more careful self-assessment and alter calibration. A three-step incentive structure is recommended for future studies to examine both behavioural and metacognitive effects.

Although overconfidence can be influenced by a wide range of socioemotional and contextual factors, the present study adopts a cognitive bias perspective, in line with the heuristics-and-biases tradition. This framing enables a focused examination of self-evaluation discrepancies without extending into broader developmental or affective mechanisms. Factors such as self-efficacy beliefs, emotional regulation, and perceived social support may also influence overconfidence among young decision makers. Future research can build upon these findings by incorporating socioemotional and developmental variables, such as emotional regulation, perceived social support, and transitional stressors, into analyses of confidence calibration in emerging adulthood.

A key limitation is that participants were students in different courses taught by the same instructor, who also designed the task. The instructor was absent during exam administration, and participation was voluntary. Of 591 eligible students, 414 responded. Despite efforts to reduce implicit pressure, future studies should use independent administration or blinding to improve data independence and validity. We scheduled the task during the final exam period to minimize priming effects from earlier classroom discussions. Although this timing could raise concerns about potential recency or availability effects, we consider these risks minimal for several reasons. First, a significant interval separated the earlier course discussions from the final exam period, reducing the likelihood that recent exposure influenced participants’ predictions. Second, the materials students reviewed before exams, such as organizational behaviour, human resource management, and introduction to business, were theoretical and unrelated to the social inference-based guessing task. Therefore, while the timing may prompt concerns about cognitive accessibility, we maintain that the study’s design and context substantially limit the relevance of such effects. Procedural steps were taken to minimize social desirability, such as ensuring voluntary participation, using independent proctors, and excluding the instructor from data collection. Still, the study’s inherent structure may have introduced subtle expectancy effects. The task was designed by the course instructor and based on personal preferences, so participants may have perceived implicit cues about expected responses. Even without direct interaction, the instructor’s dual role as educational authority and evaluator of correctness may have influenced confidence or responses. This contextual dependency, though methodologically managed as much as possible, remains a limitation for internal validity. It should be considered when interpreting the strength and generalizability of the findings.

This study did not measure musical literacy, which represents a key limitation. People with higher musical knowledge may feel more competent in such a prediction task. However, we did not seek to examine the sources of overconfidence. Rather, we aimed to empirically demonstrate its presence and forms. Because self-assessed confidence was already substantially higher than actual performance, we believe participants across all levels of expertise exhibited clear overconfidence. We encourage future studies to assess musical literacy and other individual differences to better explore underlying mechanisms.

The experimental task employed in this study did not incorporate dynamic or probabilistically defined uncertainty. Rather, the ambiguity experienced by participants was primarily subjective, resulting from limited information and the absence of feedback. Future research could utilize simulation-based or evolving decision environments to examine the interaction between objective task uncertainty and confidence judgments.

The task design in this study is grounded in the principle of parsimony. Instead of introducing artificial complexity or relying on externally validated knowledge domains, the design aims to capture subjective inference and confidence within a familiar and socially relevant context. In this respect, the task aligns with Occam’s Razor by prioritizing conceptual clarity and ecological validity over psychometric sophistication. Although the one-shot, dichotomous structure may limit statistical reliability, the simplicity of the task is argued to enhance interpretive transparency, especially in the context of naturalistic decision-making under uncertainty. Also, we acknowledge that relying on single-item estimates may increase measurement error even though difference scores are widely used in the overconfidence literature. Additionally, no correction for multiple comparisons was applied, as none of the group comparisons produced statistically significant results.

Future studies may examine how specific features of the digital environments that shape Generation Z—such as performance visibility, online comparison behaviours, or algorithmic feedback mechanisms—moderate the expression of overconfidence in decision-making contexts. It is also important to explore the effects of interventions such as calibration training, statistical intuition, and metacognitive feedback on overconfidence.

## Figures and Tables

**Table 1:** Instruments and operationalization of the study.

Item	Variable	Measured Overconfidence Dimension	Explanation
a. Guess the song	Accuracy (*either 0 or 100*)	Not a direct measure of confidence, to give a baseline level of achievement	This data indicates whether a student is genuinely successful in their guesses. When used alongside other metrics, it can be utilized to analyse overconfidence.
b. Guess your probability of guessing right	Self-Assessment (*range: 0–100*)	Overestimation	Positive gap between perceived and actual success (e.g., high confidence but incorrect answer).
		Underestimation	Negative gap between perceived and actual success (e.g., low confidence but correct answer).
		Calibration bias	Absolute difference between perceived probability and actual accuracy (regardless of direction).
c. Guess the probability of your classmates’ guessing right	(Overplacement Gap) Social Comparison (*range: 0–100*)	Overplacement	Students who expect their own success to surpass that of their peers tend to demonstrate the better-than-average effect, also known as overplacement.
c′. The actual performance per peer group	Overplacement (Moore & Healy, 2008) (*range: 0–100*)	Actual accuracy rate of the peers	Calculated as the average correct response rate across all participants (actual mean = 45%). Used as a reference value for estimating overplacement.

**Table 2:** Descriptive statistics (Mean and SD) of key variables.

Variable	General (Mean ± SD)	Female (Mean ± SD)	Male (Mean ± SD)
Self-Assessment Score (*0–100*)	63.51 ± 37.58	62.90 ± 37.08	63.99 ± 33.45
Accuracy (*0–100*)	45.41 ± 49.84	46.99 ± 50.04	44.15 ± 49.76
Peer Expectation (*0–100*)	34.61 ± 30.96	36.21 ± 33.68	33.33 ± 28.64
Overestimation Score (*−100–100*)	18.10 ± 59.69	15.90 ± 59.51	19.84 ± 59.90
Calibration Error (*0–100*)	49.73 ± 37.58	47.41 ± 39.19	51.56 ± 36.24
Overplacement Gap (−67–39)	−0.0025 ± 34.97	−0.5662 ± 36.88	0.4440 ± 33.45

**Table 3:** Accuracy distribution across confidence groups.

Confidence Group—Accuracy Crosstabulation				
		Accuracy		Total N
		0	100	
Confidence Groups	Underconfident	0	131	131
	Well-calibrated	33	57	90
	Overconfident	193	0	193
Total		226	188	414

## Data Availability

The data that support the findings of this study are available from the corresponding author upon reasonable request.
